# Circulating microRNA-422a is associated with lymphatic metastasis in lung cancer

**DOI:** 10.18632/oncotarget.15025

**Published:** 2017-02-02

**Authors:** Lina Wu, Bo Hu, Bingtian Zhao, Yinan Liu, Yue Yang, Lijian Zhang, Jinfeng Chen

**Affiliations:** ^1^ Central Laboratory, Key Laboratory of Carcinogenesis and Translational Research, Ministry of Education, Beijing Cancer Hospital & Institute, Peking University Cancer Hospital, Beijing, China; ^2^ Key Laboratory of Carcinogenesis and Translational Research, Ministry of Education, Department of Thoracic Surgery II, Peking University School of Oncology, Beijing Cancer Hospital & Institute, Beijing, China; ^3^ Department of Respiratory & Critical Care Medicine, Tianjin Chest Hospital, Tianjin, China

**Keywords:** microRNA, miR-422a, lung cancer, lymphatic metastasis, biomarker

## Abstract

To identify specific circulating microRNAs that were associated with the lymphatic metastasis in lung cancer, we performed miRNA microarray analysis of lymph node with and without metastasis from five lung cancer patients. Top six differentially expressed miRNAs were selected for further validation. A training cohort of 26 patients with lung cancer was firstly recruited and the selected miRNAs in the plasma samples were investigated. miRNA-422a, with highest diagnostic accuracy in lymphatic metastasis was identified (AUC, area under the receiver operating characteristic curve, 0.744; 95%CI, 0.570-0.918). The diagnostic value of miR-422a was also demonstrated by a validation cohort of 51 lung cancer patients (AUC, 0.880; 95%CI, 0.787-0.972). Moreover, a high diagnostic value was also observed after integrated analysis of training and validation cohorts (AUC, 0.792; 95%CI, 0.688-0.896). The odds ratio of high miR-422a expression for lymphatic metastasis in lung cancer was 13.645 (95%CI, 2.677-69.553) after adjustment of the potential confounding factors. Furthermore, we predicted the target genes of miR-422a by combining the online database, miRcords, and the data from GEO and TCGA. Sixty-one target genes of miR-422a that might be involved in lymphatic metastasis in lung cancer were identified. And GO analysis suggested multiple target genes relatively concentrated in the biological processes of apoptosis, transport, and protein phosphorylation.

## INTRODUCTION

Lung cancer is one of the leading causes of cancer related death worldwide and its 5-year survival rate is low, about 15%-20%[1]. Surgical resection is an effective approach in achieving long-term survival and widely used in lung cancer. However, postoperative relapse and subsequent lymphatic and hematogenous metastasis still result in 90% of mortalities in lung cancer [2]. Lymphatic metastasis is directly associated with distant recurrence and overall survival (OS) in resected non-small cell lung cancer [3]. The image approaches such as PET/CT are currently most widely used for lymph node staging, however, the sensitivity for detecting metastatic tumor in lymph nodes smaller than 1 cm is low [4, 5]. Therefore, searching for biomarkers that can predict early metastatic phenomenon is beneficial to patients’ survival. Molecular markers including microRNAs will be of great use for a more accurate risk assessment of nodal metastasis.

MicroRNAs (miRNAs or miRs) are a family of endogenous and small RNA gene products (19 - 25 nucleotides in length) [6]. Over 50% annotated human miRNA genes are located within the cancer-associated genomic regions or fragile sites and exert important functions similar to oncogenes or tumor suppressors in malignancy [7]. These small RNA molecules can be efficiently retrieved from (fixed or frozen) tumor samples or biological fluids and display high stability and tissue specificity [8]. Previous studies demonstrated that a number of miRNAs have potential roles in diagnosis, staging, and prediction of outcomes in various types of cancer including lung cancer [9, 10]. Until now, many miRNAs have also been identified to be correlated with lymph node metastasis of lung cancer [11].

In the present study, we performed miRNAs microarray analysis in lymph node tissues of the patients with lung cancer and screened the differentially expressed miRNAs related to lymph node metastasis. Then we enrolled a training cohort of lung cancer patients and detected the expressions of top candidate miRNAs in plasma samples. The potential diagnostic value of the selected miRNAs in distinguishing lymph node metastasis was analyzed. Furthermore, miR-422a, the miRNA with highest accuracy, was validated in another cohort. Finally, the predicted target genes of miR-422a were analyzed by bioinformatic methods.

## RESULTS

### Literature review

To summarize the miRNAs that were correlated with lymphatic metastasis in lung cancer in previous reports, we performed a systematic literature review in two English databases (PubMed and Embase) up to June 30, 2016. After initial screening by reading the article titles and abstracts, 187 articles were left for selection. 54 papers were excluded according to the inclusion criteria, of which, 28 were irrelevant studies, 12 were review articles, 8 were only experimental studies, and 8 only reported the panel or signature of multiple miRNAs. Finally, 133 papers reporting 115 miRNAs were identified. Of the 115 miRNAs, 95 miRNAs were found to be associated with lymphatic metastasis in lung cancer in at least one publication while 20 were not correlated with the metastasis (Supplementary Table 1). miR-21, 10b, 148b, 155, and 200c were the most studied spots.

### miRNA microarray analysis in NSCLC with lymphatic metastasis or not

**Figure 1:**
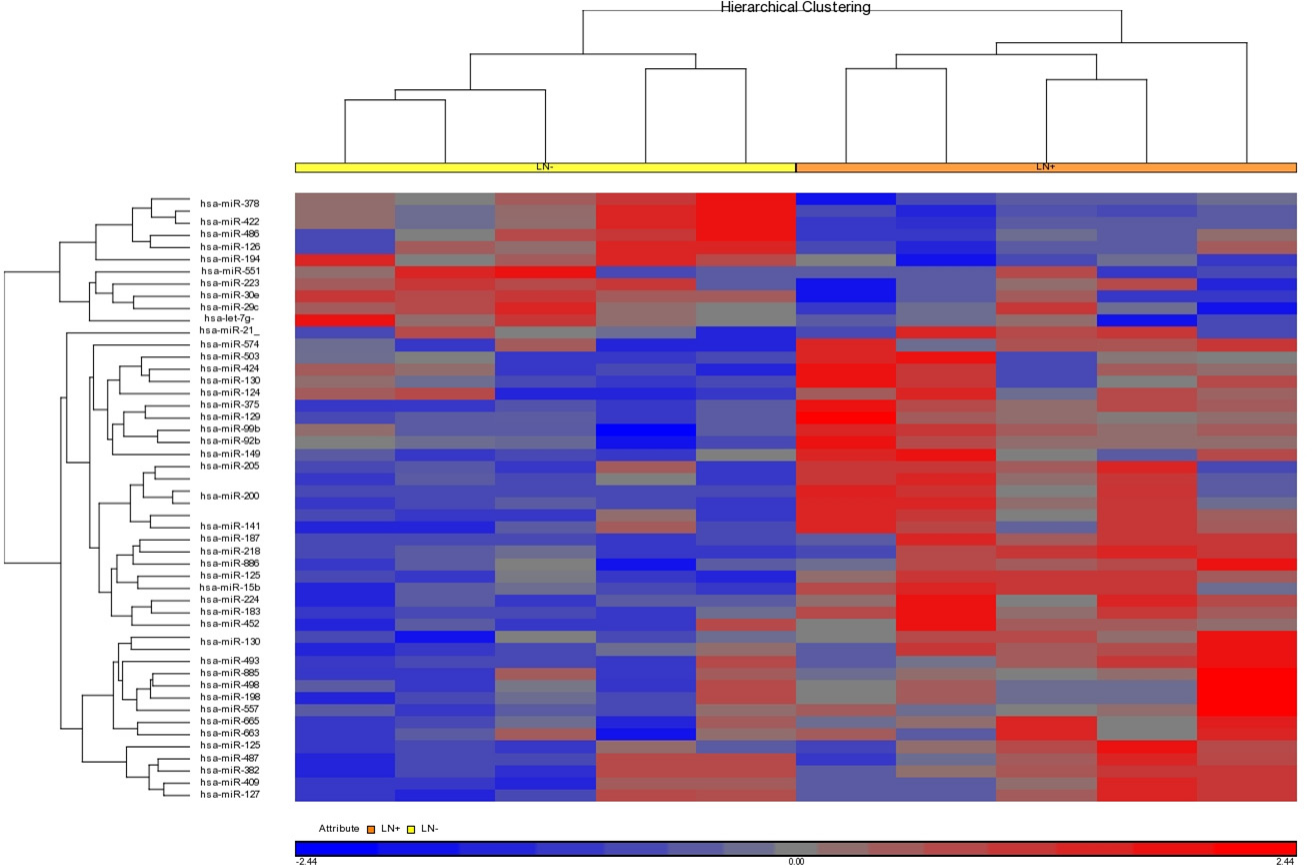
Microarray analysis of miRNAs of the metastatic and non-metastatic lymph nodes from five patients with lung cancer

In the present study, we aimed to identify novel miRNAs correlated with lymphatic metastasis in NSCLC. Firstly, we performed miRNA microarray analysis in lymph node tissues with metastasis from five lung cancer patients and compared with that in the corresponding lymph node without metastasis. After data processing and analysis, a set of 50 miRNAs were identified to be differentially expressed between metastatic lymph nodes and non metastatic lymph nodes, of which, 39 were up-regulated and 11 were down regulated (Figure 1 and Table 1). Interestingly, 18 of the 50 miRNAs were also presented in the literature review.

**Table 1:** Top 50 differentially expressed miRNAs in metastatic lymph node compared with noncancerous lymph node identified by microarray analysis in five patients with lung cancer

miRNAs	Fold change (LN+ vs LN-)	P value
**hsa-miR-375**	59.917	0.013
**hsa-miR-205**	49.518	0.014
hsa-miR-200a	40.533	0.010
**hsa-miR-183**	39.393	0.010
hsa-miR-200b	38.828	0.008
hsa-miR-200b-star	29.311	0.019
**hsa-miR-200c**	15.411	0.005
hsa-miR-187	15.292	0.013
**hsa-miR-224**	14.627	0.002
**hsa-miR-141**	13.350	0.046
hsa-miR-886-5p	9.666	0.017
hsa-miR-1290	7.783	0.050
hsa-miR-1308	7.548	0.004
hsa-miR-1244	5.058	0.031
**hsa-miR-452**	4.885	0.047
hsa-miR-149	4.395	0.033
hsa-miR-382	4.353	0.032
**hsa-miR-125a-5p**	4.336	0.004
hsa-miR-92b	4.085	0.013
**hsa-miR-503**	3.822	0.017
hsa-miR-885-3p	3.479	0.048
**hsa-miR-125b-1-star**	3.432	0.021
hsa-miR-424-star	3.355	0.008
hsa-miR-1300	3.194	0.027
hsa-miR-493	3.041	0.032
hsa-miR-99b-star	2.996	0.008
**hsa-miR-409-3p**	2.869	0.008
**hsa-miR-130b**	2.813	0.021
hsa-miR-218	2.776	0.030
hsa-miR-557	2.708	0.020
hsa-miR-127-3p	2.630	0.005
hsa-miR-15b	2.536	0.003
hsa-miR-665	2.496	0.001
hsa-miR-663b	2.388	0.024
**hsa-miR-198**	2.322	0.028
hsa-miR-498	2.290	0.046
hsa-miR-487b	2.225	0.042
hsa-miR-574-3p	2.210	0.028
**hsa-miR-21**	2.178	0.009
hsa-miR-1263	−2.034	0.039
hsa-miR-194	−2.089	0.000
hsa-miR-551b	−2.262	0.043
**hsa-let-7g-star**	−2.380	0.042
**hsa-miR-30e**	−2.892	0.008
**hsa-miR-223**	−2.911	0.048
hsa-miR-29c	−3.368	0.031
hsa-miR-378	−5.044	0.007
**hsa-miR-486-3p**	−6.148	0.012
hsa-miR-422a	−6.207	0.006
hsa-miR-378-star	−9.385	0.005

Note: LN: lymph node; Bold, miRNAs have been also identified in the previous reports

### Value of the top differential expressed miRNA in diagnoses lymph node metastasis in lung cancer

**Figure 2:**
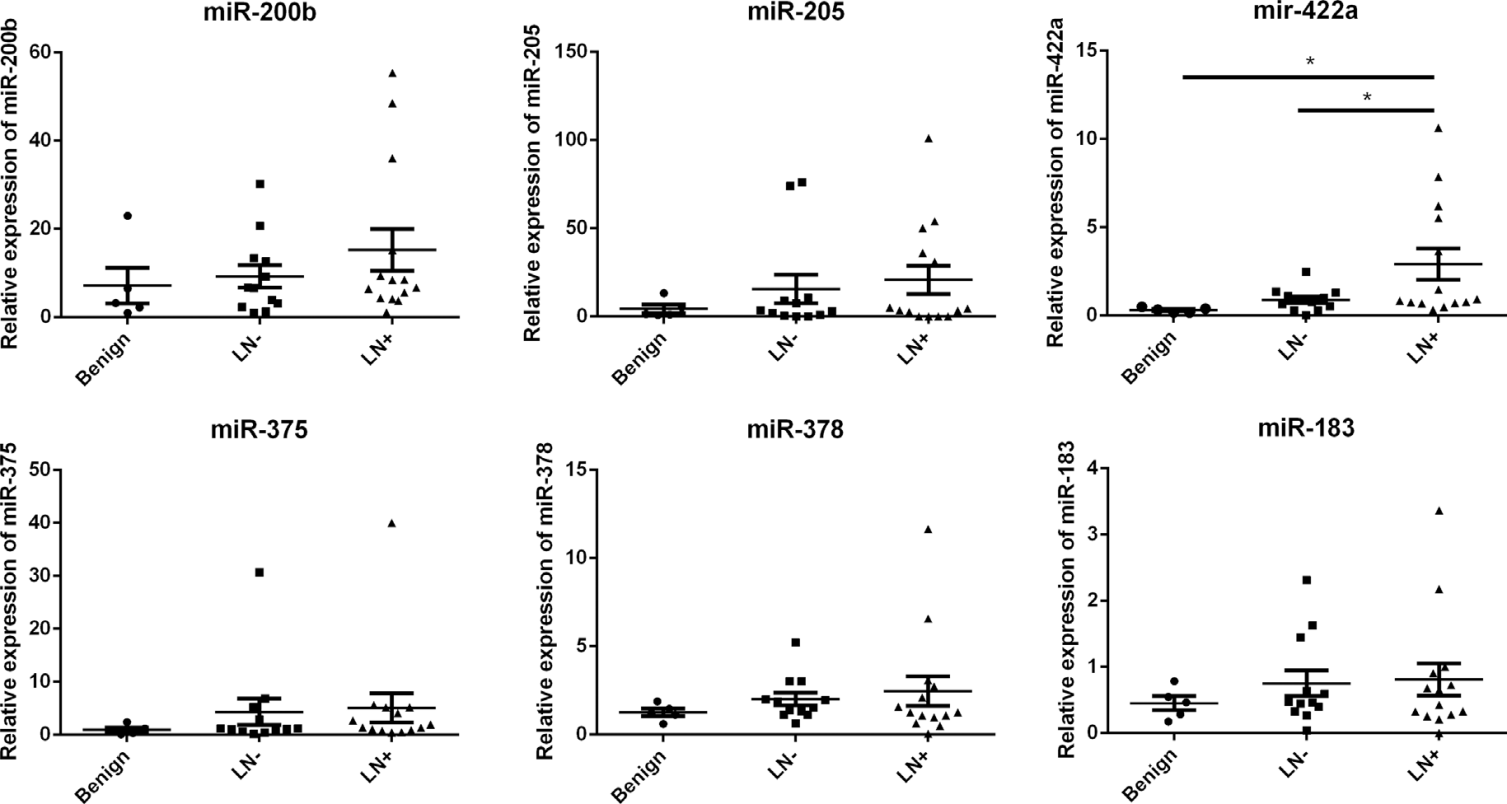
Expression of candidate miRNAs in training cohort of 26 lung cancer with or without lymphatic metastasis and five patients with benign lung disease

**Figure 3:**
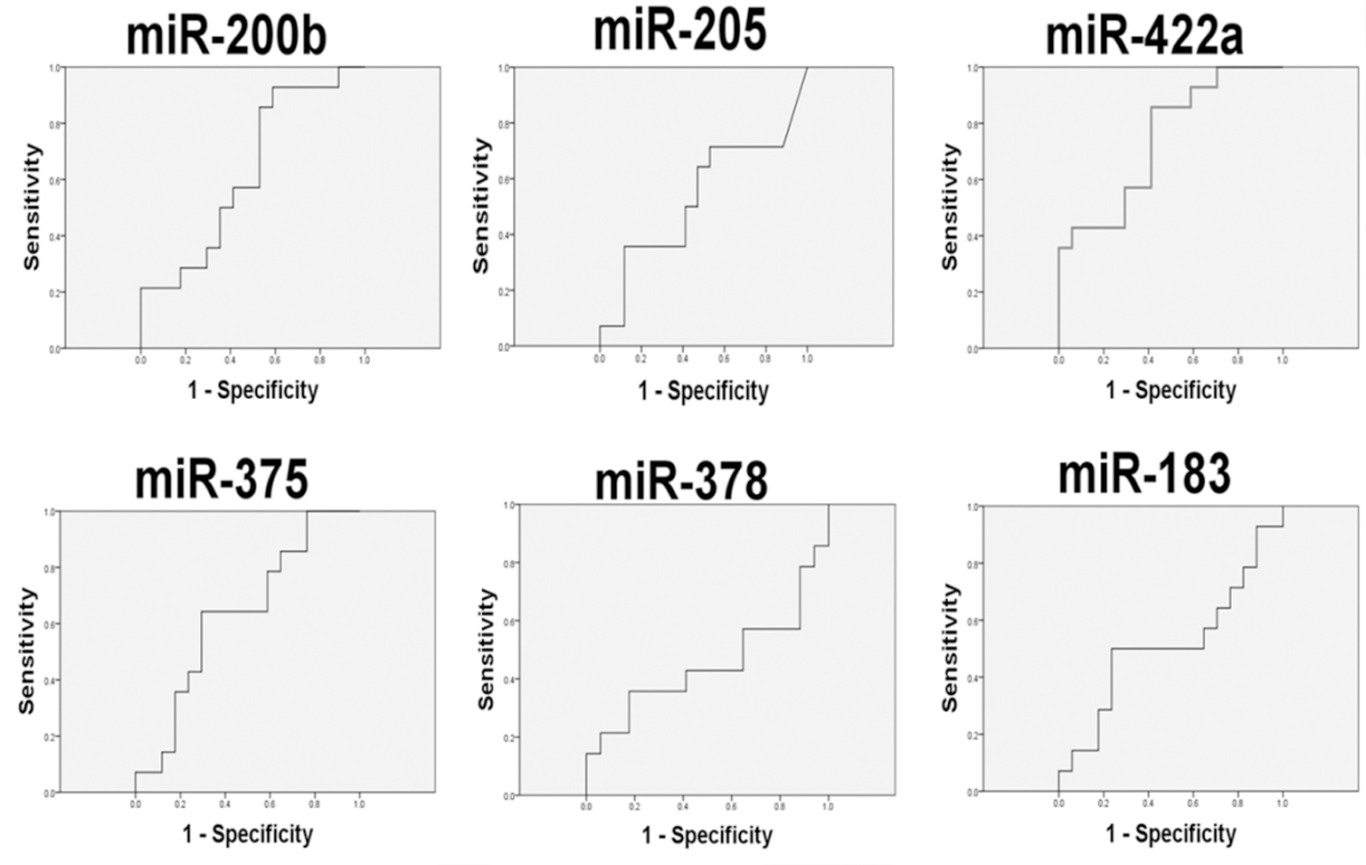
The accuracy of plasma circulating candidate miRNAs in the diagnosis of lymphatic metastasis in training cohort of 26 lung cancer patients

**Figure 4:**
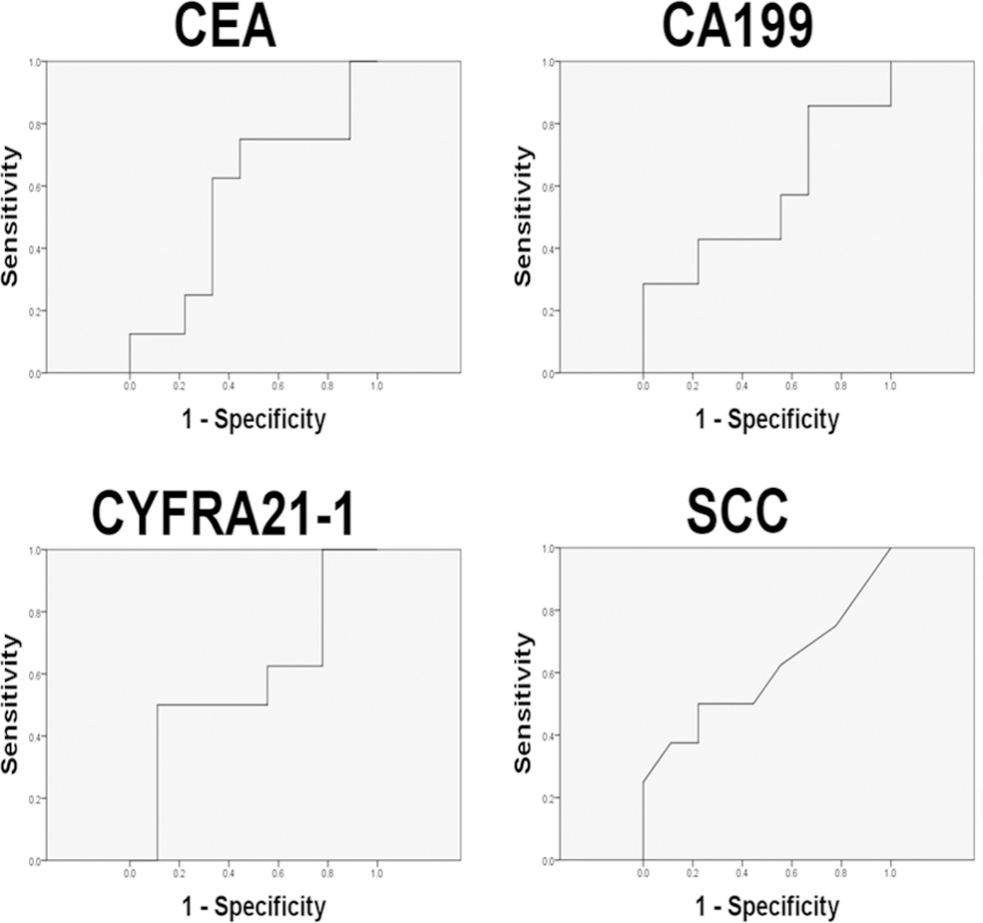
The accuracy of known tumor markers in the diagnosis of lymphatic metastasis in training cohort of lung cancer patients

To determine the diagnostic value of the miRNA identified by miRNA microarray analysis, we chose six miRNAs (hsa-miR-375, hsa-miR-205, hsa-miR-183, hsa-miR-200b, hsa-miR-378, and hsa-miR-422a). And we evaluated their expression levels in plasma samples from 26 lung cancer patients (14 with lymphatic metastasis and 12 patients without lymphatic metastasis, Table 2) and five patients with benign lung diseases by quantitative real-time polymerase chain reaction (qRT-PCR). The results suggested that all the six miRNAs were highly expressed in lung cancer with lymph node metastasis compared with the cancers without lymph node metastasis and benign diseases, of note miR-422a exhibited significant difference (Figure 2). Then Receiver Operating Characteristic curve (ROC) analysis was performed to compare the diagnosis accuracy among miRNAs or traditional tumor markers such as carcino embryonie antigen (CEA), cancer antigen 199 (CA199), cytokeratin 19 fragments (CYFRA21-1), and squamous cell carcinoma antigen (SCC). And the results indicated that the candidate miRNA, miR-422a, showed highest accuracy in predicting lymphatic metastasis with an AUC value of 0.744 (Figures 3–4 and Table 3). In addition, correlations among the six miRNAs were also analyzed. And the correlations of miR-200b with miR-183, miR-375 with miR-183 and miR-200b, and miR-422a with miR-183, miR-200b, miR-375, and miR-378 were identified (Table 4). At same time, to clarify if the aberrant expression of miR-422a in plasma were resulted from the cancer tissue, we downloaded a publically available dataset (GSE16025) from NCBI GEO database, which contained the miR-422a expression data in 20 lung cancers with lymphatic metastasis, 41 without lymphatic metastasis, and 10 normal lung tissues [12]. Consistent with our results in plasma samples, miR-422a expression was increased significantly from normal lung tissue to cancer tissue with lymphatic metastasis (Supplementary Figure 1).

**Table 2:** Clinical characteristics of the lung cancer patients

Clinicopathological features	Training cohort (n=26)	Validation cohort (n=51)
**Age** (years)	59.8±6.2	58.9±8.9
**Sex**		
Male	11	29
Female	15	22
**TNM**		
Ia	10	28
IIa	8	9
IIb	1	1
IIIa	7	6
IIIb	0	1
IV	0	6
**Tumor diameter (cm)**	2.74±1.04	3.04±1.51
**T stage**		
T1	22	33
T2	3	17
T3	1	1
**N stage**		
N0	12	40
N1	7	1
N2	7	7
N3	0	3
**M stage**		
M0	26	45
M1	0	6
**Histology**		
Adenocarcinoma	22	13
Squamous cell carcinoma	2	34
Small cell lung cancer	2	4
**Lymph node metastasis**		
Yes	14	11
No	12	40

**Table 3:** ROC curve analysis of selected miRNAs and known tumor markers in diagnosis of lymphatic metastasis in lung cancer patients

Biomarkers	AUC	95%CI	P value
**Training cohort**			
miR-183	0.513	0.296 - 0.729	0.110
miR-200b	0.630	0.431 - 0.830	0.102
miR-205	0.534	0.318 - 0.749	0.110
miR-375	0.634	0.436 - 0.833	0.101
miR-378	0.450	0.228 - 0.671	0.113
miR-422a	0.744	0.570 - 0.918	0.089
CEA	0.635	0.347 - 0.923	0.368
CA199	0.556	0.249 - 0.863	0.711
CYFRA211	0.540	0.234 - 0.845	0.791
SCC	0.548	0.241 - 0.855	0.751
**Validation cohort**			
miR-422a	0.880	0.787-0.972	0.000
**All patients**			
miR-422a	0.792	0.688-0.896	0.000

Note: CEA: carcino embryonie antigen; CA199: carbohydrate antigen-199; CYFEA211: cytokerantin-19-fragment; SCC: squamous cell carcinoma antigen

**Table 4:** Correlations among the selected miRNAs in training cohort

miRNAs	miR-183		miR-200b		miR-205		miR-375		miR-378	
	r	P	r	P	r	P	r	P	r	P
**miR-183**										
**miR-200b**	**0.699**	**0.000**								
**miR-205**	0.075	0.688	0.284	0.121						
**miR-375**	**0.549**	**0.001**	**0.682**	**0.000**	0.185	0.318				
**miR-378**	0.135	0.469	0.301	0.099	0.021	0.909	0.303	0.097		
**miR-422**	**0.369**	**0.041**	**0.554**	**0.001**	−0.019	0.921	**0.417**	**0.020**	**0.735**	**0.000**

### Validation of miR-422a in predicting lymphatic metastasis of lung cancer

**Figure 5:**
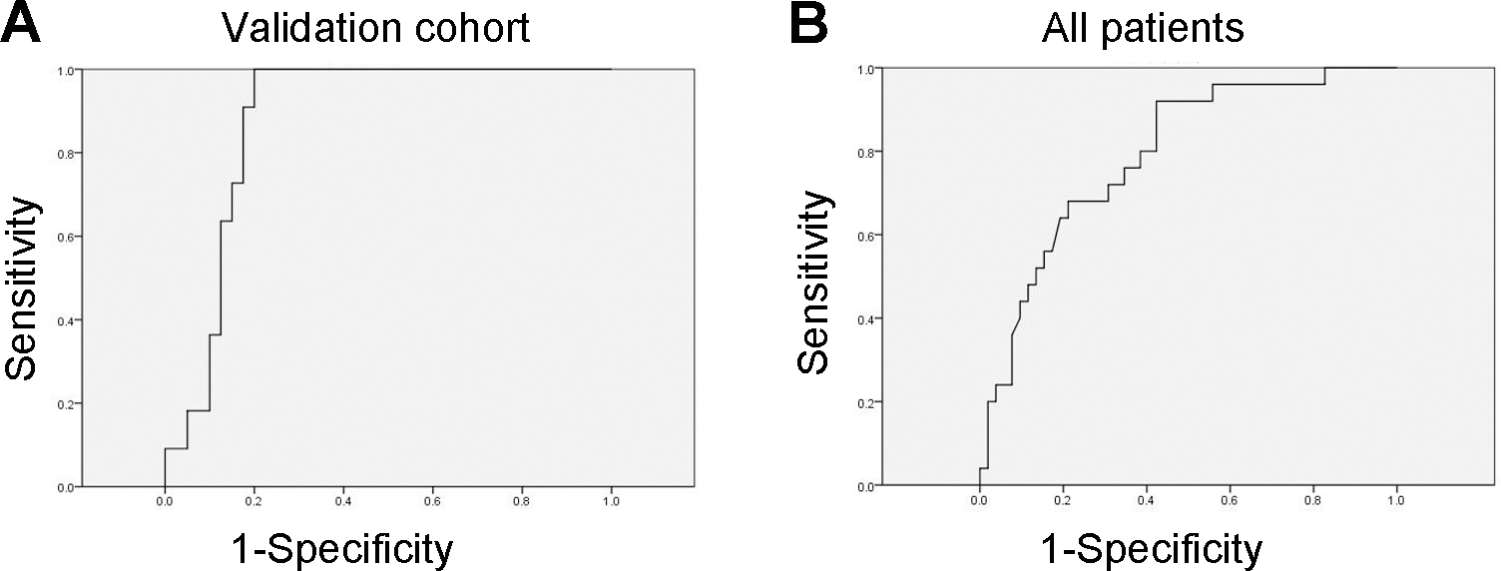
Validation of miR-422a as a biomarker for lymph nodes metastasis **A**. The accuracy of miR-422a in the diagnosis of lymphatic metastasis in validation cohort of 51 lung cancer patients. **B**. miR-422a expression data from both of training and validation cohorts were normalized and integrated. The diagnostic value of miR-422a in predicting lymphatic metastasis in all patients was analyzed.

Another cohort consisting of 40 lymphatic metastatic lung cancer cases and 11 non-lymphatic metastatic cases was used to further verify the diagnostic value of miR-422a (Table 2). The plasma miR-422a levels were determined by qRT-PCR and ROC curve analysis was performed. The expression of miR-422a in samples with lymphatic metastasis was significantly higher than those without lymphatic metastasis, consistent with the results of training cohort. ROC analysis suggested that miR-422a exhibited high diagnostic accuracy with an AUC value of 0.880 (Table 3 and Figure 5A). Furthermore, the training cohort and validation cohort were integrated by normalizing the miR-422 expression data and a high diagnostic accuracy of miR-422a was still observed (AUC=0.792, Table 3 and Figure 5B).

Meanwhile, the correlations between miR-422a and the clinical features including age, gender, histological subtypes, Tumor diameter, T stage, M stage, and the lymph node metastasis in all patients were investigated. The miR-422a expression level less than the cut-off value from the ROC analysis was defined as “Low expression” and that more than the cut-off value was defined as “High expression”. The results suggested that miR-422a was correlated with histological subtype, T stage, and tumor diameter stratified by 2.55 cm, a cut-off value from ROC analysis with lymph node metastasis as state variable in all patients with lung cancer (Table 5).

**Table 5:** Correlation of miR-422a with clinical features in all 77 patients with lung cancer

Clinical features	Low expression	High expression	P-value
**Age** (years)			
<60	15	22	0.814
≥60	16	21	
**Gender**			0.226
Male	14	26	
Female	18	19	
**Histology**			0.019
SCC	12	23	
ADC	20	16	
SCLC	0	6	
**Tumor diameter (cm)**	2.60±1.32	3.20±1.38	0.059
**Tumor diameter (cm)**			0.021
<2.55	21	17	
≥2.55	11	28	
**T stage**			0.012
T1	28	27	
T2-3	4	18	
**M stage**			
M0	31	40	0.391
M1	1	5	
**Lymph node metastasis**			
No (N0)	30	22	0.000
Yes (N1-3)	2	23	

In addition, the correlations of lymphatic node metastasis with the clinical features were also explored. The results suggested that significant correlation was observed between lymphatic metastasis and the clinical feature tumor diameter, but not age, gender, histology subtype, T stage, and M stage (Table 6). Based on the above results, histology subtype and T stage/tumor diameter might be the potential confounding factors for the association between miR-422a expression and lymphatic metastasis. After adjustment of the potential confounding factors, histology subtype and T stage, or histology subtype and tumor diameter, the odds ratios of miR-422a for lymphatic metastasis were 13.645 (95%CI, 2.677-69.553; *P*=0.002) and 11.459 (95%CI, 2.337-56.118; *P*=0.003), respectively, similar with the crude OR (15.682; 95%CI, 3.341-73.596; *P*=0.000) and indicating a strong association of miR-422a with lymphatic metastasis in lung cancer (Table 7). On the other hand, the analyses of the data from GSE16025 also revealed that miR-422a levels in cancer tissue was associated with lymphatic metastasis in lung cancer in Univariate analysis (OR, 6.500; 95%CI, 1.136-37.203, P=0.035) and multivariate analysis (adjusted OR, 8.133; 95%CI, 1.103-59.986, P=0.040) (Table 7). The cross verification between different sample source further demonstrated the association of miR-422a with lymph node metastasis.

**Table 6:** Correlation of lymphatic node metastasis with clinical features in all 77 patients with lung cancer

Clinical features	LN-	LN+	P value
**Age**			
<60	26	11	1.000
≥60	26	11	
**Gender**			
Male	27	13	0.995
Female	25	12	
**Histology**			
SCC	22	13	0.962
ADC	28	8	
SCLC	2	4	
**Tumor diameter (cm)**	2.87±1.47	3.12±1.19	0.473
**Tumor diameter (cm)**			
<2.55	31	7	0.014
≥2.55	21	18	
**T stage**			
T1	39	16	0.317
T2-3	13	9	
**M stage**			0.063
M0	50	21	
M1	2	4	
**miR-422 expression**			0.000
Low	30	2	
High	22	23	

**Table 7:** Odds ratios (ORs) of high miR-422a expression for lymphatic node metastasis in all 77 patients with lung cancer

	Crude OR	95%CI	P value	Adjusted OR	95%CI	P value	Adjusted OR	95%CI	P value
**Our study**									
miR-422a	15.682	3.341-73.596	0.000	13.645^a^	2.677-69.553	0.002	11.459^b^	2.337-56.188	0.003
**GSE16025**									
miR-422a	6.500	1.136-37.203	0.035	8.133^c^	1.103-59.986	0.040			

^a^The adjusted OR was obtained by controlling the factors of histology and T stages.

^b^The adjusted OR was obtained by controlling the factors of histology and tumor size.

^c^The adjusted OR was obtained by controlling the factors of age and T stage.

### Prediction of miR-422a target genes that might be involved in lymphatic metastasis

Given the fact that the biological significance of miRNAs deregulation relies on the actions of their targets, we aimed to identify the potential targets of miR-422a involved in lymphatic metastasis in lung cancer by prediction and data mining. Firstly, the predicted targets of the miR-422a were analyzed by the online database, miRecords and a total of 3309 predicted target genes were identified. Then, GSE51852 and GSE51853 were downloaded from NCBI, which contained both the mRNA and the miRNA expression profile of 126 lung cancer patients. 2052 genes co-expressed with miR-422a were obtained after analyzing the datasets. Thirdly, 983 lung cancer patients at N0-3 stage were downloaded from The Cancer Genome Atlas (TCGA) and 4842 differential expressed genes in lymph node metastasis cancers compared with non-metastatic cancers were identified. Finally, to find potential novel target genes of miR-422a involved in lymph node metastasis of lung cancer, the results from the above analyses were integrated and a total of 61 genes were left (Table 8). Then these genes were subjected to gene ontology (GO) analysis in the GeneCoDis3 online database (http://genecodis.cnb.csic.es). The biological processes of apoptosis, transport, and protein phosphorylation contained multiple target genes. Many of the genes were related to DNA and protein binding. And most the genes served as nucleus and membrane components (Table 9).

**Table 8:** The potential target genes of miR-422a involved in lymphatic metastasis identified by predicting by online database, miRecords and mining of the data from GEO and TCGA

Gene Symbol	Gene description
FYCO1	FYVE and coiled-coil domain containing 1
CX3CR1	chemokine (C-X3-C motif) receptor 1
PHC3	polyhomeotic homolog 3 (Drosophila)
IL33	interleukin 33
BDH2	3-hydroxybutyrate dehydrogenase, type 2
RSL1D1	ribosomal L1 domain containing 1
GOT2	glutamic-oxaloacetic transaminase 2, mitochondrial (aspartate aminotransferase 2)
FANCA	Fanconi anemia, complementation group A
PUS7	pseudouridylate synthase 7 homolog (S. cerevisiae)
ZNF362	zinc finger protein 362
PTPRE	protein tyrosine phosphatase, receptor type, E
C18orf56	chromosome 18 open reading frame 56
ARHGAP11A	Rho GTPase activating protein 11A
PTCD1	pentatricopeptide repeat domain 1
CCDC85A	coiled-coil domain containing 85A
TOB2	transducer of ERBB2, 2
SUV420H1	suppressor of variegation 4-20 homolog 1 (Drosophila)
POLR2J	polymerase (RNA) II (DNA directed) polypeptide J, 13.3kDa
ARID5B	AT rich interactive domain 5B (MRF1-like)
FANCI	Fanconi anemia, complementation group I
ARHGEF12	Rho guanine nucleotide exchange factor (GEF) 12
CISH	cytokine inducible SH2-containing protein
S1PR2	sphingosine-1-phosphate receptor 2
PCDHA9	protocadherin alpha 9
E2F2	E2F transcription factor 2
TMEM130	transmembrane protein 130
SHROOM4	shroom family member 4
TGFBR2	transforming growth factor, beta receptor II (70/80kDa)
PFKFB2	6-phosphofructo-2-kinase/fructose-2,6-biphosphatase 2
MEGF10	multiple EGF-like-domains 10
YPEL1	yippee-like 1 (Drosophila)
KIAA0247	KIAA0247
CC2D2B	coiled-coil and C2 domain containing 2B
COL9A2	collagen, type IX, alpha 2
NHLRC3	NHL repeat containing 3
APLP2	amyloid beta (A4) precursor-like protein 2
ZNF599	zinc finger protein 599
HTATIP2	HIV-1 Tat interactive protein 2, 30kDa
ATG2B	ATG2 autophagy related 2 homolog B (S. cerevisiae)
CAMKK1	calcium/calmodulin-dependent protein kinase kinase 1, alpha
C18orf1	chromosome 18 open reading frame 1
SLC16A12	solute carrier family 16, member 12 (monocarboxylic acid transporter 12)
LRRFIP2	leucine rich repeat (in FLII) interacting protein 2
JMJD6	jumonji domain containing 6
C8orf46	chromosome 8 open reading frame 46
SEZ6	seizure related 6 homolog (mouse)
ALG3	asparagine-linked glycosylation 3 homolog (S. cerevisiae, alpha-1,3-mannosyltransferase)
GPR123	G protein-coupled receptor 123
SLC34A2	solute carrier family 34 (sodium phosphate), member 2
TRDMT1	tRNA aspartic acid methyltransferase 1
ZNF552	zinc finger protein 552
RAB1A	RAB1A, member RAS oncogene family
COX4NB	COX4 neighbor
FBXO31	F-box protein 31
BTBD7	BTB (POZ) domain containing 7
SORBS1	sorbin and SH3 domain containing 1
DYRK1A	dual-specificity tyrosine-(Y)-phosphorylation regulated kinase 1A
KIAA0494	KIAA0494
JRK	jerky homolog (mouse)
CYCS	cytochrome c, somatic
ABCD4	ATP-binding cassette, sub-family D (ALD), member 4

**Table 9:** GO analysis of the potential target genes of miR-422a related to lymphatic metastasis in lung cancer

GO items	P value	Genes
**Biological process**		
Apoptotic process	0.047	E2F2, ARHGEF12, TGFBR2, CYCS, JMJD6
Transport	0.048	GOT2, ABCD4, SORBS1, CYCS, FYCO1
Protein phosphorylation	0.046	POLR2J, DYRK1A, TGFBR2, PTPRE
**Molecular function**		
DNA binding	0.031	E2F2, ARID5B, PHC3, POLR2J, ZNF552, APLP2, ZNF362, ZNF599, TRDMT1
Protein binding	0.024	E2F2, IL33, FANCA, ARID5B, POLR2J, FANCI, DYRK1A, LRRFIP2, APLP2, CX3CR1, TGFBR2, PFKFB2, HTATIP2, SORBS1, PTPRE, CYCS, FYCO1, JMJD6
**Cellular component**		
Nucleus	0.000	E2F2, FANCA, ARID5B, RSL1D1, PHC3, POLR2J, FANCI, YPEL1, ZNF552, DYRK1A, APLP2, ZNF362, ZNF599, SHROOM4, SUV420H1, HTATIP2, SORBS1, COX4NB, TOB2, PTPRE, TRDMT1, CYCS, JMJD6, CAMKK1
Integral to membrane	0.027	SLC16A12, SLC34A2, ALG3, MEGF10, C18orf1, SEZ6, APLP2, CX3CR1, GPR123, TGFBR2, ABCD4, PTPRE, KIAA0247, FYCO1, TMEM130, S1PR2, PCDHA9
Plasma membrane	0.012	SLC16A12, SLC34A2, FANCI, MEGF10, C18orf1, SEZ6, APLP2, CX3CR1, GPR123, TGFBR2, GOT2, SORBS1, PTPRE, JMJD6, S1PR2, PCDHA9

## DISCUSSION

In the present study, we screened novel circulating miRNAs in predicting lymph node metastasis in lung cancer. One novel miRNA, miR-422a, showed the highest diagnostic value compared with other miRNAs and traditional tumor markers, such as CEA, CA199, and CYFEA211. Furthermore, a validation cohort consist of 51 lung cancer patients was recruited to confirm the diagnostic value of miR-422a. ROC curve analyses revealed that miR-422a possessed a high AUC value of 0.880 with sensitivity of 86.22% and specificity of 96.55%. Some miRNAs have been reported to be correlated with lymphatic metastasis and showed in our literature review. For example, miR-196a [13] and miR-130a [14] were positively associated with lymph node metastasis, while miR-451 [15] was negatively associated with lymph node metastasis. Recent studies have focused on the sources of circulatory molecular biomarkers [16]. In previous review papers about predictive values of circulating routine plasma tumor markers, the accuracy for metastasis detection ranged from 0.815 (CYFRA 21-1) to 0.724 (CEA), respectively (AUC values) [17, 18].

miR-422a was a less studied miRNA. A few studies have revealed the involvement of miR-422a in human cancers. It is dysregulated in osteosarcoma [19], colorectal cancer [20–22], hepatocarcinoma [23], and gastric cancer [24]. miR-422a is an indicative marker for poor prognosis in osteosarcoma, gastric cancer, and hepatocarcinoma [23]. It is also associated with chemo-resistance in osteosarcoma [25]. In lung cancer, one study suggested that miR-422a can be used to classify lung adenocarcinoma from lung squamous cell carcinoma [26]. In the present study, we found that plasma miR-422a levels were upregulated in lung cancer patient with lymphatic metastasis than that in patients without lymphatic metastasis. Here, the expression changes trends of miR-422a was opposite with the results of the microarray analysis. We predicted that the sample size in microarray analysis was very small and then the individual difference led to a certain degree of bias when exploring the differential expressed microRNAs in the patients with lymphatic metastasis or not. The upregulation of plasma miR-422a in lymphatic metastatic patients were also evidenced by an independent cohort, GSE16025, which analyzing the mRNA expression profile of lung cancer tissues (Supplementary Figure 1). And our study, for first time, found that miR-422a had a potential diagnostic value in discriminating lymph node metastasis of lung cancer. To further clarify the association of miR-422a and lymphatic metastasis in lung cancer, we analyzed the associations of miR-422a with the clinical parameters and the associations of lymphatic metastasis with the clinical parameters. And the results suggested miR-422a was associated with histology and T stage/tumor diameter, which might be the confounding factors for the association of miR-422a with lymphatic metastasis. Notably, a strong significant association was still observed between miR-422a and lymphatic metastasis after adjustment of the confounding factors (Table 7). Furthermore, by analyses the mRNA profile data in cancer tissues in GSE16025, a similar association was observed between cancer tissue miR-422a level and lymphatic metastasis in lung cancer.

In summary, our findings highlight that a novel circulating miRNA, miR-422a, may serve as a non-invasive biomarker with sufficient accuracy in predicting lymph node metastasis in lung cancer patients. And the application of miR-422a in clinical practice may help for prophylactic intervention to mitigate morbidity and mortality. miR-422a may also provide a new target for therapeutic approaches in management of lung cancer.

## MATERIALS AND METHODS

### Literature review

To summary the associations of miRNAs with lymph node metastasis in lung cancer, a systematic literature search was performed independently by two authors in two databases (PubMed and EMBASE) up to June 30, 2016. The following termed were used: “lung cancer”, “lymph node OR lymph node metastasis, OR lymphatic metastasis”, and “Microrna OR miRNA”. Inclusion criteria: (1) English language papers; (2) The associations of miRNA with lymph node metastasis were determined in clinical samples; (3) single miRNA but not panel or signature of mutiple miRNAs was investigated.

### Patients and samples

Anonymized fresh metastatic lymph node samples and the compared noncancerous lymph nodes from five lung cancer patients were collected after surgical resection for miRNA profile analysis. Then, 26 lung cancer patients (14 cases with lymphatic metastasis and 12 cases without lymphatic metastasis) from August 2013 to May 2014 were included as a training cohort to determine the diagnosis value of the top differentially expressed miRNAs from miRNA profile analysis (Table 2). In addition, five patients with benign lung diseases without cancers were recruited as controls. Furthermore, another cohort of 51 lung cancer patients (LN+, 40; LN-, 11) was recruited from June 2014 to October 2015 to validate the miRNA with highest diagnosis accuracy in the training cohort (Table 2). Unlike with the microarray analysis, fresh peripheral blood samples were used. Clinicopathological characteristics of patients with lung cancer were defined according to the TNM staging system criteria of the Union for International Cancer Control. And histological diagnosis was based on the medical records. Formalin-fixed paraffin-embedded (FFPE) sections were carefully reviewed for the diagnosis of metastatic lymph nodes and noncancerous lymph nodes. All procedures were approved by the Ethics Committee of Peking University. Written informed consents were obtained from all patients or their families.

### miRNA microarray analysis

The matched frozen tissue samples of metastatic and noncancerous lymph nodes from the same patients were used to perform miRNA microarray analysis. Total RNA (100 ng) was polyA-tailed and ligated to biotinylated signal molecules using the Flash Tag™ Biotin RNA Labeling Kit (Genisphere, Llc in Hatfield, PA, USA). An enzyme-linked oligo sorbent quantitative-competitive assay was performed to verify the labeling prior to array hybridization to GeneChip® miRNA microarrays (Affymetrix, Santa Clara, CA, USA). And then hybridization, washing, staining, and scanning were conducted using Affymetrix Gene Chip® system instruments and protocols (Affymetrix, Santa Clara, CA, USA). Subsequently, the microarray image was analyzed and the intensity values were calculated by Affymetrix Gene Chip® Command Console Software version 3.0.1. Further analysis was performed using Partek Genomics Suite (Partek, St. Louis, MO, USA).

### Quantitative real-time polymerase chain reaction (qRT-PCR)

MiRNAs were extracted from the plasma samples from lung cancer patients using miRNeasy Mini Kit (Qiagen, Valencia, CA, USA) according to the manufacturer's instructions. The concentration and purity of isolated RNA was estimated using the ND-1000 microspectrophotometer (Thermo Fisher Scientific, Waltham, MA, USA). RNA integrity was assessed on BioAnalyzer 2100 using BioAnalyzer RNA 6000 Nano LabChip Kit (Agilent Technologies, Palo Alto, CA, USA). Then TaqMan miRNA assays (Applied Biosystems, Thermo Fisher Scientific, Waltham, MA, USA) were used to detect the expression levels of mature miRNAs. For reverse transcription reactions, 10 ng of total RNA was mixed with the reverse transcription primers, reacted at 16°C for 30 min, 42°C for 30 min, and 85°C for 5 min, and then maintained at 4°C. Following the reverse transcription reactions, 1 μL of cDNA was used for polymerase chain reaction (PCR) using 2 μl of the TaqMan primers. PCR reactions were conducted at 95°C for 10 min followed by 40 cycles of 95°C for 15 s and 60°C for 60 s on an ABI 7500 Fast Real-time PCR system (Thermo Fisher Scientific, Waltham, MA, USA). Real-time PCR results were analyzed and the expression of miRNA levels was calculated using the 2^−ΔΔCt^ method and normalized to miR-16, an internal reference control. The probes of the candidate miRNAs were show in Supplementary Table 2.

### Predicted targets analysis of miR-422a

The predicted targets of the miR-422a were analyzed by an online database, miRecords (http://c1.accurascience.com/miRecords/), which is a resource for animal miRNA-target interactions. The Predicted Targets component of mIRecords integrates the predicted targets of the following miRNA target prediction tools: DIANA-microT, MicroInspector, miRanda, MirTarget2, miTarget, NBmiRTar, PicTar, PITA, RNA22, RNAhybrid, and TargetScan/TargertScanS [27]. The target genes of miR-422a were derived when the target genes were predicted by at least three algorithms in mIRecords. In addition, the GEO datasets (GSE51852 and GSE51853) were downloaded from NCBI, which contained both the mRNA and the miRNA expression profile of 126 lung cancer patients and were used to find the genes co-expressed with the selected miRNA, miR-422a [28]. Furthermore, mRNA profile data of 983 lung cancer patients was downloaded from TCGA database, subgrouped by lymphatic metastasis or not, and used to find genes related to lymphatic metastasis. Then, the potential target genes of miR-422a involved in lymphatic metastasis were identified by integrated analysis of predicted targets from miRcords and data mining of GEO and TCGA. Finally, the potential targets were subjected to GO analysis in the GeneCoDis3 online database (http://genecodis.cnb.csic.es).

### Statistical analysis

Statistical analyses were performed with SPSS 16.0 (IBM, Armonk, NY, USA). Kruskall Wallis H test was used for comparing miRNAs expression among multiple groups. Receiver operating characteristic (ROC) curves were established for discriminating the patients with lymphatic metastasis or not. The area under curve (AUC) value and 95% confidence intervals (CI) were calculated to determine the specificity and sensitivity. Z-score method was used to normalize the expression data of miRNA in training and validation cohorts. Chi-square test was used to explore the associations of miR-422a and clinical features and the associations of lymphatic metastasis with clinical features in the all recruited lung cancer patients and in dataset GSE16025. The correlations among the miRNAs in training cohort and the correlations between the genes and miR-422a in GEO data were analyzed by Pearson's correlation coefficient. The mRNA expressions in lymphatic metastatic lung cancer patients compared with non-lymphatic metastatic patients in TCGA data were analyzed by Student's t test. All tests were two-sided. *P* < 0.05 was considered to be statistically significant.

## SUPPLEMENTARY MATERIALS FIGURES AND TABLES




