# Supplementary material for: Circulating microRNA-422a is associated with lymphatic metastasis in lung cancer
Source: Oncotarget. 2017 Feb 2;8(26):42173–88. doi: 10.18632/oncotarget.15025 (PMC5522058; doi:10.18632/oncotarget.15025)
Supplement: Supplementary file 1 [file oncotarget-08-42173-s001.pdf]

Circulating microRNA-422a is associated with lymphatic metastasis in lung cancer

SUPPLEMENTARY MATERIALS

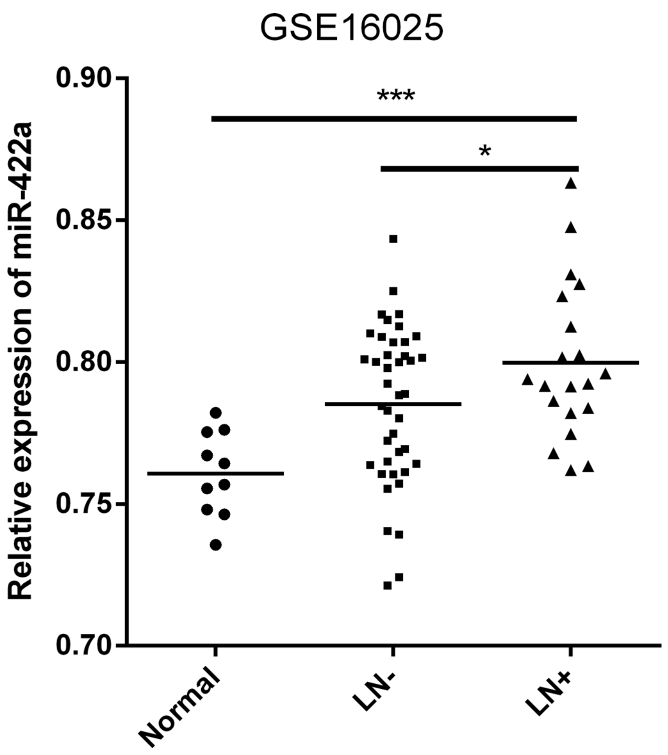

Supplementary Figure 1: miR-422a expression in normal lung tissue, and lymphatic metastatic/non-lymphatic metastatic lung cancer tissue in dataset GSE16025.

Supplementary Table 1: Summary of miRNA related with lymphatic metastasis in lung cancer in previous reports

See Supplementary File 1

Supplementary Table 2: The probe sequence of selected miRNAs

Probe set ID	Sequences
hsa-miR-375	UUUGUUCGUUCGGCUCGCGUGA
hsa-miR-183	UAUGGCACUGGUAGAAUUCACU
hsa-miR-205	UCCUUCAUUCCACCGGAGUCUG
hsa-miR-200b	UAAUACUGCCUGGUAAUGAUGA
hsa-miR-422a	ACUGGACUUAGGGUCAGAAGGC
hsa-miR-378	CUCCUGACUCCAGGUCCUGUGU
