# Supplementary material for: Circulating microRNA-422a is associated with lymphatic metastasis in lung cancer
Source: Oncotarget. 2017 Feb 2;8(26):42173–88. doi: 10.18632/oncotarget.15025 (PMC5522058; doi:10.18632/oncotarget.15025)
Supplement: Supplementary file 2 [file oncotarget-08-42173-s002.docx]

**Table 1. Summary of miRNA related with lymphatic metastasis in lung cancer in previous reports**

| **miRs** | **Down-regulation** | **Up-regulation** | **Non-significant** | **Total reference** |
| --- | --- | --- | --- | --- |
| miR-21 |  | 6 | 3 | 9 |
| mir-10b |  | 4 |  | 4 |
| miR-148b | 2 |  | 2 | 4 |
| mir-155 |  | 1 | 3 | 4 |
| miR-200c | 2 |  | 2 | 4 |
| miR-124 | 2 | 1 |  | 3 |
| miR-130a | 2 | 1 |  | 3 |
| miR-145 | 2 | 1 |  | 3 |
| miR-148a | 3 |  |  | 3 |
| miR-150 |  | 1 | 2 | 3 |
| miR-451 | 3 |  |  | 3 |
| miR-99a | 3 |  |  | 3 |
| let-7c | 2 |  |  | 2 |
| let-7e |  |  | 2 | 2 |
| miR-10a |  | 2 |  | 2 |
| miR-126 | 1 |  | 1 | 2 |
| miR-141 |  | 1 | 1 | 2 |
| miR-143 |  |  | 2 | 2 |
| miR-152 |  |  | 2 | 2 |
| miR-153 | 2 |  |  | 2 |
| miR-154 | 2 |  |  | 2 |
| miR-193a-3p | 2 |  |  | 2 |
| miR-204 | 2 |  |  | 2 |
| miR-210 |  | 1 | 1 | 2 |
| miR-221 |  | 1 | 1 | 2 |
| miR-29b | 1 | 1 |  | 2 |
| miR-32 | 2 |  |  | 2 |
| miR-338-3p |  | 1 | 1 | 2 |
| miR-34a |  |  | 2 | 2 |
| miR-365 | 2 |  |  | 2 |
| miR-375 | 1 | 1 |  | 2 |
| miR-452 | 2 |  |  | 2 |
| miR-638 | 2 |  |  | 2 |
| miR-650 | 1 |  | 1 | 2 |
| miR-9 |  | 1 | 1 | 2 |
| let-7g | 1 |  |  | 1 |
| miR-1 | 1 |  |  | 1 |
| miR-101 | 1 |  |  | 1 |
| miR-107 | 1 |  |  | 1 |
| miR-1207-5p | 1 |  |  | 1 |
| miR-1258 | 1 |  |  | 1 |
| miR-125a-35p | | 1 |  | 1 |
| miR-125a-5p |  |  | 1 | 1 |
| miR-125b | 1 |  |  | 1 |
| miR-1260b |  | 1 |  | 1 |
| miR-126-3p |  | 1 |  | 1 |
| miR-130b | 1 |  |  | 1 |
| miR-132 | 1 |  |  | 1 |
| miR-133a | 1 |  |  | 1 |
| miR-133b | 1 |  |  | 1 |
| miR-137 | 1 |  |  | 1 |
| miR-138 | 1 |  |  | 1 |
| miR-145-5p | 1 |  |  | 1 |
| miR-146a | 1 |  |  | 1 |
| miR-147 | 1 |  |  | 1 |
| miR-181a |  |  | 1 | 1 |
| miR-181b | 1 |  |  | 1 |
| miR-182 |  |  | 1 | 1 |
| miR-183-3p |  | 1 |  | 1 |
| miR-18a |  | 1 |  | 1 |
| miR-193a-5p | 1 |  |  | 1 |
| miR-196a |  | 1 |  | 1 |
| miR-197 |  | 1 |  | 1 |
| miR-1976 |  | 1 |  | 1 |
| miR-198 | 1 |  |  | 1 |
| miR-19a |  | 1 |  | 1 |
| miR-19b |  | 1 |  | 1 |
| miR-203 | 1 |  |  | 1 |
| miR-205 |  | 1 |  | 1 |
| miR-206 | 1 |  |  | 1 |
| miR-214 |  | 1 |  | 1 |
| miR-215 | 1 |  |  | 1 |
| miR-21-5p |  |  | 1 | 1 |
| miR-216a | 1 |  |  | 1 |
| miR-223 |  | 1 |  | 1 |
| miR-224 |  | 1 |  | 1 |
| miR-23a |  | 1 |  | 1 |
| miR-23a-5p | 1 |  |  | 1 |
| miR-25 |  | 1 |  | 1 |
| miR-26a |  |  | 1 | 1 |
| miR-26b |  |  | 1 | 1 |
| miR-26b-5p |  |  | 1 | 1 |
| miR-30a | 1 |  |  | 1 |
| miR-30b | 1 |  |  | 1 |
| miR-30e |  |  | 1 | 1 |
| miR-30e-3p | 1 |  |  | 1 |
| miR-30e-5p |  |  | 1 | 1 |
| miR-31 |  | 1 |  | 1 |
| miR-320 | 1 |  |  | 1 |
| miR-339-5p | 1 |  |  | 1 |
| miR-345 | 1 |  |  | 1 |
| miR-34b | 1 |  |  | 1 |
| miR-361-3p | 1 |  |  | 1 |
| miR-363-3p | 1 |  |  | 1 |
| miR-3940-5p |  |  | 1 | 1 |
| miR-409-3p | 1 |  |  | 1 |
| miR-423-3p | 1 |  |  | 1 |
| miR-449a | 1 |  |  | 1 |
| miR-454 |  | 1 |  | 1 |
| miR-486 |  |  | 1 | 1 |
| miR-486-5p | 1 |  |  | 1 |
| miR-491-5p | 1 |  |  | 1 |
| miR-494 |  | 1 |  | 1 |
| miR-499 |  |  | 1 | 1 |
| miR-503 | 1 |  |  | 1 |
| miR-5100 |  |  | 1 | 1 |
| miR-520a-3p |  | 1 |  | 1 |
| miR-5481 | 1 |  |  | 1 |
| miR-623 |  |  | 1 | 1 |
| miR-652-3p |  | 1 |  | 1 |
| miR-663a | 1 |  |  | 1 |
| miR-675-5p | 1 |  |  | 1 |
| miR-708-5p | 1 |  |  | 1 |
| miR-761 |  | 1 |  | 1 |
| miR-92a |  | 1 |  | 1 |
